# Affected Other Prevalence and Profiles: Findings from a Cross-Sectional Australian Population-Representative Gambling Study

**DOI:** 10.1007/s10899-025-10377-z

**Published:** 2025-02-19

**Authors:** Nicki A. Dowling, Kimberley Spence, Matthew Browne, Matthew Rockloff, Stephanie S. Merkouris

**Affiliations:** 1 School of Psychology, Deakin University, Geelong, Australia; 2 School of Health, Medical and Applied Sciences Central, Queensland University, 6 University Dr, Branyan, Bundaberg, QLD 4670 Australia

**Keywords:** Problem gambling, Affected others, Mental health, Help-seeking, Prevalence, Gambling harm

## Abstract

Gambling-related harm can extend to family members and friends but few population-representative studies have investigated affected other (AO) prevalence estimates and profiles in the general population. Using data from the 5000 adult respondents in the Fourth Social and Economic Impact Study of Gambling in Tasmania, this study aimed to: (1) identify prevalence estimates of AO status and professional help-seeking; (2) establish the socio-demographic and gambling profiles of AOs; (3) extend the growing literature examining negative mental health characteristics experienced by AOs, after accounting for socio-demographic characteristics and other potential sources of harm; and (4) explore the degree to which gender moderates these relationships. Results found that 1 in 20 adults (5.11%, 95% CI: 4.33, 6.01) reported past-year AO status but only 1 in 7 AOs (14.15%, 95% CI: 9.01, 21.52) had ever sought help in relation to another person’s gambling (i.e., < 1% of all adults). AOs were significantly more likely than non-AOs to be younger, Australian-born, employed, and living in households with children. They were significantly more likely than non-AOs to report depression symptoms, anxiety symptoms, binge drinking, tobacco use, and drug use, even after controlling for socio-demographics and other potential sources of harm. Finally, they were more likely to report their own gambling participation, problems, and harm but only 2.20% (95% CI: 0.69, 6.78) had ever sought help for their own gambling. These findings suggest that a considerable proportion of AOs in the general population may benefit from support to improve their own mental health and address their own gambling harm.

## Background

The public health perspective highlights the need to consider harm not only to people who gamble, but also to their family members and friends (Shaffer & Korn, 2002). Self-reports from gamblers suggest that one person’s gambling problems negatively affects at least six others, while low- and moderate-risk gambling impacts one and three others, respectively (Goodwin et al., 2017). Internationally, the prevalence of affected others (AOs) in the population ranges from 2.0 to 21.2% based on AO self-report (Castrén et al., 2021; Hing et al., 2022; Lind et al., 2022; Salonen et al., 2014, 2015, 2016; Shiue, 2015; Svensson et al., 2013; Wenzel et al., 2008). Most studies are conducted in Nordic countries, such as Finland (12.9–21.2%) (Castrén et al., 2021; Lind et al., 2022; Salonen et al., 2014, 2015, 2016), Norway (2.0%) (Wenzel et al., 2008), and Sweden (18.2%) (Svensson et al., 2013), with only single studies conducted in Australia (6.0%) (Hing et al., 2022) and Japan (4.3%) (Shiue, 2015).

Variability in AO prevalence estimates can be attributed to measurement factors, including the relationship types considered, measurement timeframe, and thresholds of impact. Broad definitions of AO status involving any significant others (6.0–21.2%) yield higher estimates than narrower definitions focusing on family members (2.0–10.0%) or non-family members/close friends (4.6–13.7%). Lifetime estimates of AO status (18.2–21.2%) exceed current estimates (6.0–12.9%), with similar trends for family members (2.0–10.0% lifetime cf. 4.3–5.6% current) and non-family members/close friends (12.4–13.7% lifetime cf. 4.6% current). Moreover, the lowest estimate (2.0%) was derived from a restrictive multiple-item measure of affected family member status (Wenzel et al., 2008). Finally, it is important to distinguish exposure to gambling problems from experiencing gambling-related harm, given that many people exposed to gambling problems by their significant others (34.7–58.3%), family members (29.6–43.6%), non-family members (67.0%), and close friends (35.8%) report no harm (Castrén et al., 2021; Lind et al., 2022; Salonen et al., 2016). The exception is an Australian current estimate of 6.0% based on respondents being “personally affected by another person’s gambling”, although this estimate excluded land-based-only gamblers (Hing et al., 2022).

## Socio-Demographic Profiles

Population-level studies demonstrate mixed regarding AO socio-demographic profiles. AO prevalence estimates among all significant others range from 13.7 to 20.6% for women and 12.1 to 21.8% for men, with most studies reporting no gender differences (Hing et al., 2022; Lind et al., 2022; Salonen et al., 2014, 2016; Tulloch et al., 2021). Some studies, however, have found women (Castrén et al., 2021; Tulloch et al., 2021, 2023) or men (Svensson et al., 2013) were more likely to be AOs. Women (3.9–11.9%) are more likely than men (1.1–6.8%) to be affected family members and men (5.7–16.2%) are more likely than women (3.7–11.2%) to be affected non-family members or close friends (Castrén et al., 2021; Lind et al., 2022; Salonen et al., 2014, 2016; Wenzel et al., 2008). Younger people are more likely to be AOs (including affected family members and close friends) (Hing et al., 2022; Lind et al., 2022; Svensson et al., 2013; Tulloch et al., 2021, 2023; Wenzel et al., 2008), with one exception identifying no age differences (Salonen et al., 2014).

Population-level data on other socio-demographic characteristics is even more inconsistent (Hing et al., 2022; Salonen et al., 2014; Svensson et al., 2013; Tulloch et al., 2021, 2023; Wenzel et al., 2008), likely due to variability in AO measurement across studies (Dowling et al., 2021, 2025a). The only study measuring harm rather than exposure (Hing et al., 2022) found AOs in Australia were more likely to have never been married, in defacto relationships, and divorced or separated; and less likely to be currently married or widowed. AOs were also more likely to be Australian-born but speak a language other than English at home, with no differences on education or income. Other population-level studies suggest AO status may be associated with living with children (Salonen et al., 2014; Svensson et al., 2013), retirement (Tulloch et al., 2021), or reliance on social welfare (Svensson et al., 2013).

## Gambling Behaviour Profiles

Population-level studies consistently demonstrate that AOs engage in higher rates of gambling than non-AOs. AO status, including affected close friends, has been associated with higher past-year gambling participation (Hing et al., 2022; Lind et al., 2022), although one study found this only for women (Salonen et al., 2014). AOs, including affected family members and close friends are also more likely to report past-year low-risk, moderate-risk, and problem gambling (Hing et al., 2022; Lind et al., 2022; Tulloch et al., 2021, 2023; Wenzel et al., 2008), although some studies found this for both genders (Salonen et al., 2014) or for men only (Svensson et al., 2013).

## Negative Mental Health Characteristic Profiles

Gambling-related harm refers to any negative consequence from gambling that reduces health or wellbeing at individual, family, community, or population levels (Langham et al., 2016). Like gamblers, AOs report gambling harms across emotional, financial, relationship, health, work or study, cultural, and criminal domains (Dowling et al., 2021, 2025a; Langham et al., 2016). These harms have been identified using measures that do not directly reference gambling as the cause (non-attributable harm) and those that directly reference gambling (attributable harm). Studies assessing non-attributable harm using standardised measures, which demonstrate the least consistent evidence of gambling-related harm, rely on small samples of treatment-seeking AOs, limiting generalisability to the broader population (Dowling et al., 2021, 2025).

The Stress–Strain-Coping-Support model (SSCS) (Orford et al., 2005, 2010) suggests that chronic gambling-related stress in families leads to strain in the form of reduced health and wellbeing for family members. Population-level studies using both standardized and non-standardized tools that do not explicitly reference gambling demonstrate that AOs are more likely than non-AO samples to report negative mental health characteristics, such as mental health problems (i.e., psychological distress), depression, hazardous drinking, and substance abuse (Salonen et al., 2014; Svensson et al., 2013; Wenzel et al., 2008), albeit with one equivocal finding for anxiety (Wenzel et al., 2008). Some evidence suggests gendered patterns, linking AO status to smoking daily in only women and risky alcohol consumption in only men (Salonen et al., 2014).

These studies often fail to control for co-occurring risk factors, potentially conflating gambling-related harm with other sources, such as other negative mental health characteristics (Dowling et al., 2021, 2025a; Quilty et al., 2015). Studies controlling for these factors demonstrate that most associations between AO status and negative mental health characteristics remain significant, although sometimes attenuated. For example, Svensson et al. (2013) found that associations between AO status and psychological distress remained significant for both genders after adjusting for age and problem gambling severity. However, hazardous alcohol use remained significant for men but not women in the second wave of their study after including these covariates. Similarly, Salonen et al. (2014) found psychological distress was no longer significant for AOs after accounting for socio-demographics, gambling, and wellbeing, but smoking daily persisted for female AOs and risky alcohol consumption persisted for male AOs. Several studies have also found mixed evidence for associations after controlling for socio-demographics and other potential sources of harm without first conducting unadjusted analyses. For example, Lind et al. (2022) found AO status, including affected family members, was associated with psychological distress and risky alcohol consumption after controlling for socio-demographic, gambling, and wellbeing. In contrast, Shiue (2015) found no mental illness differences between affected and non-affected family members after controlling for age and sex.

Taken together, these studies provide mixed evidence on whether associations between AO status and negative mental health outcomes persist after controlling for other potential sources of harm. They also offer limited insight into which factors explain any attenuation of these associations. Additionally, most studies have been conducted in Nordic countries, focusing on exposure to gambling problems rather than harm. Further research is therefore required to explore the negative mental health characteristics of AOs in non-Nordic, population-representative samples, controlling for various sources of harm.

## Help-Seeking Profiles

Although AOs report high rates of gambling-related harm, few studies have explored their help-seeking behaviours (Dowling et al., 2021, 2025b). The SSCS model suggests that professional and informal support can influence the strain AOs experience (Orford et al., 2005, 2010). In Sweden, only 8.5% of male AOs and 10.9% of females AOs in the general population sought help or information relating to another person’s gambling, with no gender differences (Svensson et al., 2013). To date, no study has identified the proportion of AOs seeking help for their own gambling, highlighting this as an important focus for research.

## Study Aims

An enhanced understanding of AO prevalence and profiles can inform the development of more effective prevention and intervention efforts (Dowling et al., 2009, 2021, 2025a; Estevez et al., 2020; Salonen et al., 2014). Using a large population-representative sample from the Australian state of Tasmania that employed a measure of current experience of gambling harms resulting from another person’s gambling, the aims of this exploratory study were to: (1) identify prevalence estimates of AO status and professional help-seeking; (2) establish the socio-demographic and gambling profiles of AOs; (3) extend the growing literature examining negative mental health characteristics (non-attributable gambling harms) experienced by AOs, after accounting for socio-demographic characteristics and other potential sources of harm, such as problem gambling severity and other negative mental health characteristics; and (4) explore the degree to which gender moderates the relationships between AO status and negative mental health characteristics.

## Method

### Participants and Procedure

Data from the Fourth Social and Economic Impact Study (SEIS) of Gambling in Tasmania (ACIL Allen Consulting et al., 2017) were subject to secondary data analysis. Data were collected from 5000 Tasmanian adults via Computer Assisted Telephone Interviewing (CATI) surveys conducted between June 13 and August 7, 2017. Because the Fourth SEIS focused on state-level estimates, a stratified approach based on broad geographic regions, proportional to the population, was employed. A dual-frame sampling approach was adopted, with a 50% allocation for landline phones (n = 2493) and a 50% allocation for mobile phones (n = 2507). The sample was drawn from three sources: random digit dial (RDD) landline, pre-screened RDD mobile phone numbers, and listed mobile phone numbers. Consistent with previous SEIS surveys (ACIL Allen et al., 2014; Allen Consulting Group et al., 2011), landline respondents were selected using the “youngest male aged 18 years and over” method (or the “youngest female method aged 18 years or over” method if no males were available). This method of respondent selection was undertaken to overcome a known historical bias, whereby females are over-represented via landline sample frames compared to population benchmarks and mobile phone sampling frames (Dowling et al., 2015; Jackson et al., 2014). By preferentially selecting for males, a more balanced gender profile for the whole sample was possible. While it introduces some bias, it effectively reduces a more important bias of unequal gender distribution. In contrast, consistent with previous SEIS surveys (ACIL Allen et al., 2014), mobile phone respondents were defined as any person who answered the phone, was a Tasmanian resident, and was 18 years of age or older.

A pilot test of 50 interviews was first conducted to check the quality of the survey. A total of 90,315 calls were made to 26,476 sample records to achieve 5000 CATI interviews. The survey’s final combined response rate, calculated using the American Association for Public Opinion Research (AAPOR) Response Rate 3 method, which allocates records as either in-scope or out-of-scope based on the distribution of records with a known call outcome (American Association of Public Opinion Research, 2011), was 41.5%.

A two-stage weighting approach was employed: first, a design weight was calculated to adjust for the probability of being sampled by landline and mobile phone; second, this weight was adjusted through iterative proportion fitting to align with population benchmarks for telephone status, age, sex, region, education, and country of birth. The average interview duration was 15 min.

The Fourth SEIS received ethics approval from Deakin University’s Human Research Ethics Committee (protocol #2017-145), and the secondary analysis presented in this manuscript was approved by an exemption from ethical review provided by Deakin University’s Human Research Ethics Committee (project 2020-410).

### Measures

Respondents were required to provide information relating to AO status, socio-demographic characteristics, gambling characteristics (gambling consumption indices, problem gambling severity, gambling-related harm), negative mental health characteristics (depression symptoms, anxiety symptoms, binge drinking, tobacco use, and drug use), and help-seeking characteristics as detailed below.

### AO Status

A single item with a binary response option was employed to determine past-year AO status: *In the past 12 months, have you been personally affected by another person’s gambling?*

### Socio-Demographic Characteristics

Demographic characteristics included gender, age, household structure, employment status, annual gross personal income, country of birth, main language spoken at home, highest level of education, and postcode. The Index of Relative Socioeconomic Advantage and Disadvantage (IRSAD) (Froelicher, 2017; Statistics, 2021) was employed to estimate socioeconomic status based on the residential postcode. Lower scores on the index indicate relatively higher levels of disadvantage.

### Gambling Characteristics

#### Gambling Consumption Indices

Past-year gambling participation was measured across nine gambling activities: electronic gaming machines, horse or greyhound racing, instant scratch tickets, lotteries, keno, casino table games, bingo, sporting or other events, and informal private games. Other gambling consumption indices (frequency, expenditure, and duration) were collected for each endorsed gambling activity. For gambling frequency items, typical wording was: ‘*In the last 12 months, how many times per week, per month or per year have you played/bet on [gambling activity]?’* for different modalities (e.g. venue, telephone, racetrack, off-course venue, internet on mobile device, internet using desktop computer). Annual gambling frequency was calculated by standardising each response to an estimated yearly frequency then summing these yearly frequencies across gambling activities. For gambling expenditure items, typical wording was: ‘*In the past 12 months, approximately how much money, on average, did you spend during each session of [gambling activity]?’*. Total annual gambling expenditure was calculated by multiplying gambling frequency with session expenditure estimates for each activity then summing these yearly gambling expenditures across all gambling activities. For gambling duration items, typical wording was: “*In the past 12 months, how much time on average (in minutes) did you spend playing/betting on [gambling activity] during each visit/ session/transaction of [gambling activity]?”.* Total annual gambling duration was calculated by multiplying gambling frequency with session duration estimates for each gambling activity then summing these yearly gambling durations across all gambling activities.

#### Problem Gambling Severity

Past-year problem gambling severity was measured using the nine-item Problem Gambling Severity Index (PGSI) of the Canadian Problem Gambling Index (CPGI) (Ferris & Wynne, 2001). Respondents indicated the frequency with which each item applied to them over the previous 12 months on a four-point scale: (0) *never*, (1) *sometimes*, (2) *most of the time*, and (3) *almost always*. PGSI scores range from 0 to 27, with higher scores indicating greater problem gambling severity. These scores can be used to categorise gambling as non-problem gambling (score of 0), low-risk gambling (scores of 1 or 2), moderate-risk gambling (scores between 3 and 7), or problem gambling (scores of 8 or higher). The PGSI has demonstrated strong psychometric properties, including good internal consistency, test–retest reliability, criterion validity with gambling involvement measures, unitary dimensional structure, item variability, and concurrent validity (Ferris & Wynne, 2001; McMillen et al., 2007; Neal et al., 2005). It has also displayed excellent sensitivity (0.62–0.83) and specificity (1.00) with reference to other measures of problem gambling severity (Ferris & Wynne, 2001).

#### Gambling-Related Harm

The 10-item Short Gambling Harms Scale (Browne et al., 2017) was employed to measure harm attributed to one’s own gambling. Designed as a population-level measure of past-year gambling-related harm to monitor the impact of gambling on the community, the SGHS was derived from a pool of 73 specific harms associated with problem gambling. The selection of the ten items was guided by an algorithm aiming to maximise sensitivity and cover a broad range of constructs. Each item offers a binary response option, and total scores are determined by summing the endorsed items. The SGHS exhibits a high correlation (0.94) with the 72-item checklist and demonstrates a minimal percentage of false negatives (4.8%) in identifying individuals harmed by gambling. It demonstrates robust reliability (α = 0.93; coefficient omega = 0.83), homogeneity, and unidimensionality. Higher SGHS scores are associated with problem gambling severity, gambling consumption, lower quality of life, and symptoms of addictive gambling.

### Negative Mental Health Characteristics

#### Depression Symptoms

Screening for depression used the Physical Health Questionnaire-2 (PHQ-2) (Kroenke et al., 2003). This concise two-item screening tool includes the first two items of the Physical Health Questionnaire, thereby capturing the core diagnostic symptoms for major depressive disorder. Respondents were asked to assess the frequency of each item over the preceding two weeks, using a 4-point scale ranging from (0) *not at all* to (3) *nearly every day*. Scores on the PHQ-2 range from 0 to 6, with scores of 3 or higher indicating a positive screen for major depressive disorder (Kroenke et al., 2003). Compared to the Structured Clinical Interview for DSM-III-R (SCID), the PHQ-2 has demonstrated good sensitivity (0.83) and specificity (0.90) for classifying major depression. This scale has also demonstrated good to excellent internal consistency (α = 0.77–0.83) (Ahn et al., 2019; Carey et al., 2016; Maroufizadeh et al., 2019; Plummer et al., 2016).

#### Anxiety Symptoms

The Generalised Anxiety Disorder-2 (GAD-2) (Kroenke et al., 2007) was employed to measure symptoms of generalised anxiety. This brief screening tool comprises the first two items of the Generalised Anxiety Disorder (GAD) questionnaire which represent the core diagnostic symptoms for generalised anxiety disorder. Respondents were prompted to evaluate the frequency of each item over the preceding two weeks, using a 4-point scale ranging from (0) *not at all* to (3) *nearly every day*. Scores range from 0 to 6, with scores of 3 or above indicating a positive screen for generalised anxiety disorder (Kroenke et al., 2007). Compared to Structured Clinical Interview for DSM-IV (SCID), the GAD-2 has demonstrated good sensitivity (0.76–0.93) and specificity (0.80–0.85). This scale has also demonstrated excellent internal consistency (α = 0.81–0.86) (Ahn et al., 2019; Plummer et al., 2016; Staples et al., 2019).

#### Binge Drinking

Binge drinking was measured using the AUDIT-3, which is the third item of the Alcohol Use Disorders Identification Test (AUDIT) (Babor et al., 2001). The modified response options for this item were: (1) every day; (2) 4–6 times a week; (3) 2–3 times a week; (4) once a week; (5) 2–3 times a month; (6) monthly or less; (7) not in the last year; and (8) never. The AUDIT-3 has demonstrated good sensitivity (0.77–0.90) and specificity (0.53–0.77) compared to the AUDIT (Gual et al., 2002).

#### Tobacco and Drug Use

Single items were employed to measure tobacco use, illegal drug use (cannabis or other non-prescription substances such as cocaine, amphetamine type stimulants, inhalants, hallucinogens, heroin), and misuse of prescription medication (sleeping pills, pain medications, or diet pills) in the previous 12 months. These items were based on a single-item screening test for drug use in primary care, which has demonstrated excellent sensitivity (0.86–0.96) and specificity (0.89–0.96) in detecting past year drug use compared to the Composite International Diagnostic Interview (Smith et al., 2010). In this study, response categories were (1) everyday; (2) 4–6 times a week; (3) 2–3 times a week; (4) weekly; (5) 2–3 times a month; (6) monthly or less and (7) not in the last 12 months. Endorsement of options 1 to 6 on either illegal drug use or misuse of prescription medication was categorised as past-year drug use.

### Help-Seeking Characteristics

Two single items with binary response options were employed to measure lifetime gambling professional help-seeking for someone else’s gambling (*Have you ever tried to get any sort of help from the 24 h hotline, Gambler’s Help, or Gambling Help Online for problems related to someone else’s gambling?*) and their own gambling (*Have you ever tried to get any sort of help from the 24 h hotline, Gambler’s Help, or Gambling Help Online for problems related to your gambling?*).

### Data Analysis

Statistical analyses were conducted using Stata version 13 (StataCorp, 2013), employing complex survey analysis to accommodate the survey design. Prevalence estimates for current AO status and lifetime professional help-seeking (with 95% confidence intervals [CI]) were derived, broken down by gender. Missing data was high for annual gross income (16.36%) so this variable was removed from subsequent analyses. The handling of the missing data for other variables (0.00–4.08%) involved simple imputation via mean substitution for scales with less than 30% missing within a given scale (e.g., PGSI). Owing to non-normal distributions, clinical cut-off scores were applied to dichotomise all negative mental health measures.

Binary logistic and linear regression analyses, using robust estimators were carried out to investigate whether AO status predicted socio-demographic characteristics, gambling characteristics, and negative mental health characteristics (*p* < 0.05). The analysis for socio-demographic profiles were unadjusted. The analysis for gambling profiles consisted of two models: (1) unadjusted (Model 1), and (2) adjusted for socio-demographic characteristics (Model 2). The analysis for negative mental health profiles consisted of four models: (1) unadjusted (Model 1), (2) adjusted for socio-demographic factors (Model 2), (3) adjusted for socio-demographic factors and PGSI problem gambling severity (Model 3), and (4) adjusted for socio-demographic factors, PGSI problem gambling severity, and other negative mental health characteristics (Model 4). Main language spoken at home were not included as covariates in these analyses due to small cell sizes. Additionally, a series of unadjusted moderated binary logistic regression analyses explored whether gender moderated the relationships between AO status and negative mental health characteristics. The magnitude of effect sizes was interpreted as small (OR = 0.59 and 1.68; β =  < 0.20), medium (OR = 0.29 and 3.47; β = 0.20–0.49), or large (OR = 0.14 and 6.71; β =  ≥ 0.50) based on established criteria (Acock, 2008; Fey et al., 2023).

## Results

### Prevalence Estimates of AO Status and Help-Seeking

Table 1 displays the current AO status and lifetime professional help-seeking estimates, broken down by gender. Overall, the prevalence estimate of current AOs in the general population was 5.11% (95% CI: 4.33, 6.01), with no significant gender difference identified in this estimate. Overall, 14.15% (95% CI: 9.01, 21.52) of AOs had ever sought help in relation to another person’s gambling, with women significantly more likely to seek help for another person’s gambling than men. Finally, 2.20% (95% CI: 0.69, 6.78) of AOs had ever sought help in relation to their own gambling, with no significant gender difference identified in this estimate.

**Table 1:** Population prevalence estimates of current AO status and lifetime professional help-seeking^a^

	Males (*n* = 2444)^b^	Females (*n* = 2513)^b^	Total sample (*n* = 4959)
AO status	4.59% (3.60, 5.84)	5.60% (4.49, 6.98)	5.11% (4.33, 6.01)
Professional help-seeking			
Another person’s gambling	6.52%^e^ (2.62, 15.34)	20.19%^f^ (12.07, 31.80)*	14.15%^c^ (9.01, 21.52)
Own gambling	3.71%^e^ (0.90, 14.02)	1.01%^g^ (0.14, 7.02)	2.20%^d^ (0.69, 6.78)

^a^Based on weighted data. ^b^Excludes 2 participants who did not identify as male or female gender. ^c^Based on n = 202. ^d^Based on n = 203. ^e^Based on n = 93. ^f^Based on n = 109. ^g^Based on n = 110

*Significant at *p* < 0.05

### Socio-Demographic Profiles

Table 2 displays the socio-demographic profile of the sample, broken down by AO status. AO status was significantly associated with age, whereby respondents aged 65 + years were significantly less likely to be AOs than respondents aged 18–24, 25–44, and 45–64 years (*p* < 0.001). AO status was also significantly associated with household structure, whereby respondents living in households with children and other households were more likely to be AOs than respondents living in households with no children (*p* < 0.001). AOs were also more likely to be employed and born in Australia than non-AOs. However, significant effect sizes were all small magnitude (OR = 0.57–2.04). AO status was not associated with gender, annual gross personal income, English as the main language spoken at home, highest level of education, or socio-economic status.

**Table 2:** Logistic and linear regression analyses of AO status predicting sociodemographic characteristics^a^

Sociodemographic characteristics (%)	Non-AOs (*n* = 4755)	AOs (*n* = 204)	Total sample (*n* = 4959)	OR/B [95% CI]
Gender (male) (%)^b,c^	49.12%	43.93%	48.86%	OR 1.23 [0.87, 1.74]
Age (%)				OR 0.57 [0.43, 0.75]***
18–24 years	10.90%	13.34%	11.03%	
25–44 years	29.62%	39.84%	30.14%	
45–64 years	34.90%	37.52%	35.03%	
65 + years	24.58%	9.30%	23.80%	
Household structure (%)^b^				OR 1.84 [1.33, 2.56]***
Without children in household	49.54%	35.18%	48.81%	
Children in household	41.23%	50.32%	41.70%	
Other	8.33%	14.50%	8.65%	
Employment status (employed) (%)^b,d^	54.34%	64.97%	54.88%	OR 1.16 [1.13, 2.30]**
Annual gross personal income (≤ median) (%)^b,e^	62.22%	62.81%	62.25%	OR 1.33 [0.91–1.94]
Country of birth (Australia) (%)^b^	85.29%	92.35%	85.65%	OR 2.04 [1.23–3.41]**
Main language spoken at home (English) (%)	98.24%	98.46%	98.25%	OR 1.14 [0.32–4.02]
Highest level of education (%)^b,f^				OR 0.90 [0.68–1.19]
Low (less than compulsory education)	22.00%	22.36%	22.01%	
Medium (compulsory education only)	61.01%	63.86%	61.15%	
High (beyond compulsory education)	16.21%	13.07%	16.05%	
Socioeconomic status, M (SD)^g^	2.03 (1.19)	1.86 (1.13)	2.03 (1.18)	B −0.18 [−0.37, 0.02], β = 0.03

*OR* odds ratio, *B* unstandardised coefficient, *β* standardized coefficient, *95% CI* 95% confidence interval

^a^Based on weighted data. ^b^Percentages do not add to 100% due to missing data. ^c^Excludes 2 participants who did not identify as male or female gender. ^d^Employed full-time, part-time, casual, or self-employed. ^e^Median income = $57,200 (Australian Bureau of Statistics, 2016a)—citation reflects year of data collection. ^f^Education categories are consistent with Tasmanian Government guidelines (Tasmanian Government, 2023). ^g^Determined using residential postcode and matched to the Index of Relative Socio-economic Advantage and Disadvantage (IRSAD) from 2016 census data (Australian Bureau of Statistics, 2016b) to reflect year of data collection. Higher scores indicate more advantage

Significant at *p < 0.05, **p < 0.01, ***p < 0.001

### Gambling Behaviour Profiles

Table 3 displays the gambling profile of the sample, broken down by AO status. Even after controlling for socio-demographic characteristics (Model 2), AO status was significantly associated with problem gambling severity, whereby respondents with low-risk gambling (*p* = 0.009), moderate-risk gambling (*p* = 0.015), and problem gambling (*p* < 0.001) were significantly more likely to be AOs than non-gambling/non-problem gambling respondents. Moreover, respondents with problem gambling were significantly more likely to be AOs than respondents with low-risk gambling (*p* = 0.001) and moderate-risk gambling (*p* = 0.020). Compared to non-AOs, AOs were also significantly more likely to report gambling participation and any harms from their own gambling in the previous year, even after controlling for socio-demographic characteristics (Model 2). Significant effect sizes were all of small magnitude (OR = 1.67–2.58). There were no associations between AO status and other gambling consumption indices, including frequency, expenditure, and duration.

**Table 3:** Logistic and linear regression analyses of AO status predicting gambling characteristics^a^

Gambling characteristics (%)	α	Non-AO (*n* = 4755)	AO (*n* = 204)	Total sample (*n* = 4959)	OR/B [95% CI]—Model 1	aOR/B [95% CI]—Model 2	
Gambling consumption indices							
Gambling participation (%)^b^	NA	57.63%	71.15%	58.32%	OR 1.84 [1.25, 2.71] **		aOR 1.67 [1.12, 2.49]**
Gambling frequency	NA	24.18 (60.89)	28.76 (60.78)	24.41 (60.89)	B 4.58 [−5.36, 14.52], β = 0.02		aB 3.67 [−6.41, 13.75], β = 0.01
Gambling expenditure ($AUD)	NA	598.71 (2,494.81)	1,036.23 (3,518.89)	621.10 (2,558.43)	B 437.52 [−119.33, 994.37], β = 0.04		aB 386.78 [−173.47, 947.03], β = 0.03
Gambling duration (mins)	NA	705.89 (5,376.30)	1,055.56 (4,555.39)	723.73 (5,337.52)	B 349.67 [−181.45, 880.79], β = 0.01		B 135.05 [−300.26, 570.35], β = 0.01
PGSI Problem gambling severity (%)^b^	0.86				OR 2.29 [1.36, 3.84]**		aOR 2.07 [1.20, 3.57]**
Non-problem gambling		93.14%	86.33%	92.79%			
Low-risk gambling		4.56%	7.48%	4.71%			
Moderate-risk gambling		1.33%	2.31%	1.38%			
Problem gambling		0.45%	3.31%	0.59%			
SGHS Gambling harm (%)^b^	0.81	5.07%	13.06%	5.47%	OR 2.81 [1.72, 4.60]***		aOR 2.58 [1.53, 4.36]***

*α*  Cronbach’s alpha reliability coefficient, calculated based on unweighted data. NA indicates insufficient number of items to calculate alpha. *OR* odds ratio, *B* unstandardised coefficient, *β* standardised coefficient, *95% CI* = 95% confidence interval

^a^Based on weighted data. ^b^Percentages do not add to 100% due to missing data

^Significant at *p < 0.05, **p < 0.01, ***p < 0.001^

^Model 1: unadjusted model; Model 2: adjusted for gender, age, household structure, employment status, country of birth, highest level of education, and socioeconomic status^

^SGHS: Short Gambling Harm Screen; PGSI: Problem Gambling Severity Index^

### Negative Mental Health Characteristic Profiles

Table 4 displays the negative mental health characteristic profile of the sample, broken down by AO status. AO status was significantly associated with GAD-2 anxiety symptoms and tobacco use in all four models. AO status was significantly associated with PHQ-2 depression symptoms, AUDIT-3 binge drinking, and drug use in Models 1 to 3, but these associations attenuated to non-significance after controlling for other negative mental health characteristics (Model 4). Significant effect sizes were all small magnitude (OR = 1.66–3.28).

**Table 4:** Logistic regressions analyses of AO status predicting negative mental health characteristics^a^

Negative mental health characteristic (%)	Non-AOs (*n* = 4755)	AO (*n* = 204)	Total sample (*n* = 4959)	OR [95% CI]—Model 1	aOR [95% CI]—Model 2	aOR [95% CI]—Model 3	aOR [95% CI]—Model 4
PHQ-2 depression symptoms	12.78%	24.69%	13.39%	2.24 [1.47, 3.39]***	2.29 [1.45, 3.62]***	2.09 [1.31, 3.36]**	1.04 [0.53, 2.04]
GAD-2 anxiety symptoms	15.48%	37.73%	16.61%	3.27 [2.27, 4.72]***	3.28 [2.22, 4.86]***	3.06 [2.05, 4.57]***	2.76 [1.62, 4.68]***
AUDIT-3 binge drinking	15.48%	26.11%	16.02%	1.93 [1.27, 2.93]**	1.83 [1.20, 2.80]**	1.66 [1.06, 2.61]*	1.32 [0.81, 2.14]
Tobacco use	18.29%	36.62%	19.23%	2.59 [1.78, 3.75]***	2.20 [1.49, 3.26]***	2.07 [1.38, 3.11]***	1.82 [1.12, 2.95]*
Drug use	9.83%	18.46%	10.27%	2.07 [1.28, 3.36]**	1.93 [1.19, 3.11]**	1.83 [1.12, 3.00]*	1.33 [0.81, 2.18]

*OR* odds ratio, *95% CI* = 95% confidence interval

^a^Based on weighted data

Significant at *p < 0.05, ** *p* < 0.01, ***p < 0.001

Model 1: unadjusted model, Model 2: adjusted for socio-demographic characteristics (gender, age, household structure, employment status, country of birth, highest level of education and socioeconomic status), Model 3: adjusted for socio-demographic characteristics and PGSI problem gambling severity, Model 4: adjusted for socio-demographic characteristics, PGSI problem gambling severity and other negative mental health characteristics (PHQ-2 depression symptoms, GAD-2 anxiety symptoms, AUDIT-3 binge drinking, tobacco use, and drug use [excluding the negative mental health characteristic being predicted in each analysis])

*PHQ* patient health questionnaire-2, *GAD-2* Generalised anxiety disorder-2, *AUDIT-3* alcohol use disorders identification test-3

Table 5 reveals no significant interactions (*p* < 0.01) between AO status and gender when predicting negative mental health characteristics in unadjusted analyses.

**Table 5:** Logistic regression analyses of the relationship between AO status and negative mental health characteristics moderated by gender^a,b^

Negative mental health characteristics	OR	SE	*p* *value*	95% CI
PHQ-2 Depression symptoms	0.64	0.27	0.292	0.28, 1.47
GAD-2 Anxiety symptoms	0.69	0.26	0.316	0.33, 1.43
AUDIT-3 Binge drinking	1.05	0.47	0.920	0.43, 2.54
Tobacco use	1.55	0.60	0.256	0.73, 3.30,
Drug use	0.48	0.25	0.164	0.17, 1.35

*OR* odds ratio, *SE* standard error; 95% CI = 95% confidence interval

Significant at *p < 0.05, ** *p* < 0.01, ***p < 0.001

^a^Based on weighted data. ^b^All relationships moderated by gender, excluding 2 participants who did not identify as male or female gender

*PHQ-2* patient health questionnaire-2, *GAD-2* generalised anxiety disorder-2, *AUDIT-3* alcohol use disorders identification test consumption-3

## Discussion

This study sought to enhance our understanding of people affected by another person’s gambling by examining their prevalence and profiles in the Australian general population. It is the first study to estimate the prevalence of AOs who have been personally affected by another person’s gambling and their professional help-seeking in a population-representative sample, as well as to explore whether AO status predicts negative mental health characteristics after separately controlling for socio-demographic characteristics and other potential sources of harm.

### Prevalence Estimates of AO Status

The prevalence of AOs in the general population was 5.11%, indicating that a considerable proportion of the adult population may require support in relation to harm arising from another person’s gambling. This estimate, however, is at the lower end of the estimates available in the published population-representative literature in different jurisdictions (Castrén et al., 2021; Hing et al., 2022; Lind et al., 2022; Salonen et al., 2014, 2015, 2016; Shiue, 2015; Wenzel et al., 2008), even when only considering estimates of current AO status (6.0–12.9%). Although it was derived using a broad definition of AO status involving any significant others, this relatively low estimate is likely due to not only to differences in the underlying rate of AOs across jurisdictions, but also to the measurement of specific symptoms of harm, rather than exposure to another person’s gambling problem. This estimate is similar to that of the only other available Australian estimate derived from using the same harms measure in a sample that excluded land-based-only gamblers (Hing et al., 2022). Taken together, these findings highlight the need for the consistent use of a rigorous measure of current AO status measuring concrete symptoms of gambling-related harm rather than exposure to gambling problems or whether one is “affected” by someone else’s gambling, in general population surveys to facilitate comparisons across jurisdictions and over time (Dowling et al., 2021, 2025a).

### Socio-Demographic Profiles

Given the equivocal findings of past research, this study sought to clarify the socio-demographic profiles of AOs in Australia. Consistent with most past research, including that from Australia (Hing et al., 2022; Lind et al., 2022; Salonen et al., 2014, 2016; Svensson et al., 2013; Tulloch et al., 2021), younger people were more likely to report being an AO, and no gender effects were observed. Similar to some previous population-level research (Hing et al., 2022; Salonen et al., 2016; Svensson et al., 2013; Tulloch et al., 2021), those who were born in Australia, lived in a household with children, and were employed were more like to be AOs. While seemingly counterintuitive, it may be that certain cultural, social, and environmental factors related to growing up in Australian society, such as exposure to a cultural acceptance of gambling, high accessibility to gambling, and social pressure to participate in gambling activities, may influence the severity of gambling problems and harms experienced by their family members or friends. Similarly, AOs who are living in households with children and are employed may face various stressors and challenges in balancing work and childcare responsibilities with the demands of coping with their family member or friend’s gambling problem, such as increased responsibilities and stress, limited time for self-care, job performance difficulties, managing the emotional impact on children, and social isolation. Although these findings suggest that public health initiatives, at least in Australia, should target younger, Australian-born, employed people living in households with children, the small extant literature and the mixed available evidence suggests that further research examining the socio-demographic profiles of AOs using population-level data with more consistent measurement of AO status is required.

### Gambling Behaviour Profiles

Consistent with past research (Hing et al., 2022; Lind et al., 2022; Salonen et al., 2014; Svensson et al., 2013; Tulloch et al., 2021, 2023; Wenzel et al., 2008), the findings of this study suggest that AOs are more likely to gamble themselves, have gambling problems, and experience harm from their own gambling. While the association between AO status and problem gambling severity is consistent with an emerging literature, the findings from the current study extend previous findings by indicating that this association is retained after controlling for socio-demographic characteristics. In contrast, this is the first study to examine the association between AO status and harm from their own gambling, which is important given that problem gambling severity (behavioural dependence and adverse consequences) and gambling-related harm (adverse consequences only) are distinct (but highly correlated) constructs (Browne & Rockloff, 2020). Despite these associations, however, only 2% of AOs had ever sought help in relation to their own gambling. These findings have implications for gambling harm service providers in terms of targeting their services to AOs, as well as screening and treating gambling harm in the AOs attending their services using best-practice psychological interventions, including cognitive-behavioural treatments, motivational interviewing, and third-wave interventions (Cowlishaw et al., 2012; Maynard et al., 2018; Pfund et al., 2023; Yakovenko et al., 2015). They also highlight the need for future research evaluating non-attributable harm in AOs to account for problem gambling as a potential source of harm other than another person’s gambling.

## Negative Mental Health Characteristic Profiles

Consistent with most previous population-representative research (Salonen et al., 2014; Svensson et al., 2013; Wenzel et al., 2008), AOs were significantly more likely to report all negative mental health characteristics, including depression symptoms, anxiety symptoms, binge drinking, tobacco use, and drug use. Gender did not moderate these relationships, suggesting that they apply to both male and female AOs. Taken together, these findings support the need for AO treatments to target the mental health and wellbeing of the AOs themselves. However, given preliminary evidence that most AOs request interventions focusing on the needs of their gambling family member or friend rather than their own needs, there is a clear need to develop blended interventions in which AOs can concurrently enhance their self-efficacy to support gambling behaviour change, but can also be encouraged to focus on improving their own health and wellbeing (Rodda et al., 2020). To date, however, the evidence base evaluating AO interventions across the addictions is limited and suggests that AO interventions have the potential to improve some, but not all, gambler, AO, and relationship outcomes (Archer et al., 2020; Merkouris et al., 2020). The findings of this study can therefore inform the development of new interventions that specifically target the mental health and wellbeing of AOs. The high rates of symptoms for internalising and substance use disorders demonstrated by the AOs in this study suggest that these interventions include evidence-based therapies for these disorders, including cognitive-behaviour therapy, motivational interviewing, interpersonal psychotherapy, contingency management, problem-solving therapy, psychodynamic therapy, psychoeducation, and third-wave interventions, such as mindfulness-based cognitive therapy and acceptance and commitment therapy, are recommended (APS, 2018; Cuijpers et al., 2011; Cuijpers et al., 2014; Health, 2013; Minozzi et al., 2016; NICE, 2011, 2022; Pompoli et al., 2016).

Consistent with previous research (Svensson et al., 2013), all these relationships remained significant even after controlling for socio-demographic characteristics and problem gambling severity, suggesting that gambling problems or other co-occurring risk factors in AOs do not account for the worse mental health outcomes observed. However, AO status appears to predict anxiety symptoms and tobacco use independent of other negative mental health characteristics. In contrast, the associations between AO status and depression symptoms, binge drinking and drug use attenuated to non-significance in the final models, suggesting that other negative mental health characteristics accounted, at least in part, for these symptoms. These findings are consistent with previous research suggesting that some associations remained significant after controlling for socio-demographic, gambling, and health and wellbeing characteristics remained significant, while others attenuated (Salonen et al., 2014; Svensson et al., 2013). These findings highlight the need for future research exploring harms that do not specifically reference the gambling of another person control for negative mental health characteristics as other potential sources of harm. This research could reasonably be supplemented by research exploring the negative mental health characteristics that AOs themselves attribute to another person’s gambling.

### Professional Help-Seeking

Despite the considerable harm experienced by AOs, only 1 in seven (14.15%) reported they had sought professional help in relation to another person’s gambling. This estimate is somewhat higher than the only other available estimate of help-seeking, which was identified a decade ago in Sweden (Svensson et al., 2013). This finding may suggest that help-seeking by AOs is somewhat higher in Australia than Sweden or has slightly increased over time. Alternatively, given that a considerable proportion of people who are exposed to the gambling problems of their significant others do not experience any gambling-related harm (Castrén et al., 2021; Lind et al., 2022; Salonen et al., 2016), this finding may simply be an artefact of the current research measuring AO status in terms of the actual experience of gambling-related harm rather than exposure to gambling problems, per se. Regardless, access to professional support by AOs is substantially lower than expected, given they are more prevalent in the population than people experiencing harm from their own gambling but comprise less than 40% of people seeking professional help (Bastiani et al., 2015; Potenza et al., 2001; Rodda & Lubman, 2014; Rodda et al., 2013; Wood & Griffiths, 2007). Taken together, these findings highlight the need to overcome barriers to AOs seeking professional support, such as shame and stigma, and increase their awareness of gambling help services (Hing et al., 2013; Rodda et al., 2020; Svensson et al., 2013). Moreover, in this study, female AOs were significantly more likely to seek professional help than male AOs, suggesting there is a need to enhance gambling service utilisation among men specifically (Sagar-Ouriaghli et al., 2019). Low-intensity digital interventions, such as online- or app-delivered interventions, may have the potential to reach AOs who would not otherwise receive professional help (Dowling et al., 2021, 2025b).

### Study Limitations

Several limitations should be considered in interpreting the findings of this study. Firstly, the study’s cross-sectional design and lack of temporal sequencing hinder the ability to infer causation. Secondly, although the brief screening tools employed in this study exhibit good psychometric properties, they are non-diagnostic and may not fully capture the range of negative mental health characteristics present in psychiatric diagnostic categories (Ahn et al., 2019; Carey et al., 2016; Carver, 1997; Maroufizadeh et al., 2019; Plummer et al., 2016; Staples et al., 2019). Thirdly, it is critical to acknowledge that the secondary data used in this study were collected in 2017, potentially limiting its representativeness, especially in light of evolving gambling modalities (e.g., online gambling) and contextual events such as the COVID-19 pandemic. Finally, there is a possibility of experiment-wide Type I errors resulting from multiple statistical analyses. Given the adoption of a more conservative *p* value would limit the ability to identify attenuations in significance after controlling for other potential sources of harm, the few significant *p* values between 0.01 and 0.05 identified in this study should be cautiously interpreted. Taken together, these limitations underscore the need for post-COVID research employing longitudinal study designs and gold-standard instruments to measure non-attributable harms to confirm the prevalence and profiles of people experiencing harm related to another person’s gambling.

## Conclusion

This study sought to enhance our understanding of AOs by estimating their prevalence and profiles in the Australian general population. The findings revealed that 1 in 20 people in the general population had been personally harmed by another person’s gambling in the previous year but that only 1 in 7 had ever sought professional help in relation to another person’s gambling. AOs, who were most likely to be younger, Australian-born, employed people living in households with children, were significantly more likely than non-AOs to report a range of negative mental health characteristics, including depression symptoms, anxiety symptoms, binge drinking, tobacco use, and drug use, even after controlling for socio-demographics and other potential sources of harm. Taken together, these findings suggest that a considerable proportion of the adult population may benefit from support in relation to harm arising from another person’s gambling and that they require support to improve their own mental health and wellbeing in addition to enhancing their self-efficacy to support their family member or friend to change their gambling behaviour. Moreover, gambling harm services would benefit from screening and treating AOs for their own gambling problems, given their high rates of gambling participation, problems and harm and low rates of help-seeking for their own gambling.

## Data Availability

The data that support the findings of this study are not publicly available. The data are, however, available from the authors upon reasonable request and contingent upon approval from the funding body.
